# Field Efficacy and Transmission of Fast- and Slow-Killing Nucleopolyhedroviruses that Are Infectious to *Adoxophyes honmai* (Lepidoptera: Tortricidae)

**DOI:** 10.3390/v7031271

**Published:** 2015-03-18

**Authors:** Maho Takahashi, Madoka Nakai, Yasumasa Saito, Yasushi Sato, Chikara Ishijima, Yasuhisa Kunimi

**Affiliations:** 1 United Graduate School of Agricultural Science, Tokyo University of Agriculture and Technology, Fuchu, Tokyo 183-8509, Japan; E-Mails: m.tkhsh188@pref.chiba.lg.jp (M.T.); saitoyassu@gmail.com (Y.S.); kunimi@cc.tuat.ac.jp (Y.K.); 2 Chiba Prefectural Agriculture and Forestry Research Center, Chiba 266-0006, Japan; 3 NARO Institute of Vegetable and Tea Science, Shimada, Shizuoka 428-8501, Japan; E-Mails: lucifer@affrc.go.jp (Y.S.); cishiji@affrc.go.jp (C.I.); 4 NARO Agricultural Research Center, 3-1-1 Kannondai, Tsukuba, Ibaraki 305-8666, Japan

**Keywords:** nucleopolyhedrovirus, tea field, killing speed, transmission rate, leaf damage, alternative agent

## Abstract

The smaller tea tortrix, *Adoxophyes honmai* (Lepidoptera: Tortricidae), is an economically important pest of tea in Japan. Previous work showed that a fast-killing nucleopolyhedrovirus (NPV) isolated from *A. orana* (AdorNPV) and a slow-killing NPV isolated from *A. honmai* (AdhoNPV) are both infectious to *A. honmai* larvae. Field application of these different NPVs was conducted against an *A. honmai* larval population in tea plants, and the control efficacy and transmission rate of the two NPVs were compared. The slow-killing AdhoNPV showed lower field efficacy, in terms of preventing damage caused by *A. honmai* larvae against the tea plants, than the fast-killing AdorNPV. However, AdhoNPV had a significantly higher horizontal transmission rate than AdorNPV. These results show that AdorNPV is suitable as an inundative agent, while AdhoNPV is an appropriate inoculative agent.

## 1. Introduction

*Adoxophyes honmai* (Lepidoptera: Tortricidae), the smaller tea tortrix, is an economically important pest of tea in Japan. *A. honmai* usually occurs together with the tea tortrix, *Homona magnanima* (Lepidoptera: Tortricidae), another tea pest of lesser importance in Southern Japan. *A. honmai* generally has four generations per year, but five in the southern part of Japan [1].

Baculoviruses are a well-known group of insect viruses that have potential as microbial insecticides against pests in agriculture and forestry [2]. The family *Baculoviridae* comprises the four genera, which comprise *Alphabaculovirus* (nucleopolyhedrovirus (NPV), infecting lepidopteran host), *Betabaculovirus* (granulovirus (GV), infecting lepidopteran host), *Deltabaculovirus* (dipteran NPV), and *Gammabaculovirus* (hymenopteran NPV). Baculoviruses produce occlusion bodies (OBs), which are structures that embed virus particles in infected host cells and protect them from environmental hazards including ultraviolet light, thus, allowing the occluded virus particles to persist for prolonged periods between their release from an infected host and uptake by a new host. Horizontal transmission of baculoviruses from one host to another occurs when a susceptible insect ingests food contaminated with OBs [3]. *A. honmai* and *H. magnanima* are susceptible to *Adoxophyes orana* GV (AdorGV) and *H. magnanima GV* (HomaGV), respectively. A biopesticide based on both viruses has been registered as the commercial product Hamaki-Tenteki (Arysta LifeScience Corp., Tokyo, Japan). Hamaki-Tenteki is effective against *A. honmai* and *H. magnanima* but its killing speed in *A. honmai* larvae is slow: approximately 40 days are required to kill neonate larvae infected by AdorGV and HomaGV [4,5].

Development of baculovirus resistance in the codling moth, *Cydia pomonella* (Lepidoptera: Tortricidae), against commercially applied *C. pomonella* GV (CpGV) has occurred in Europe [6], but the resistance was overcome by other isolates of CpGV, which have different genotypes [7]. From this point of view, alternative virus isolates should be kept in reserve to sustain prolonged viral control of tea pests in Japan. For this purpose, Japanese isolates of two other viruses infectious to *A. honmai* larvae, *A. honmai* NPV (AdhoNPV) [8,9], and *A. honmai* entomopoxvirus (AHEV) [10], are good candidates. *A. orana* NPV (AdorNPV) was isolated in England and is closely related to AdhoNPV but its killing speed is faster than AdhoNPV [11]. If the biological characteristics of these other viruses are as suitable as AdorGV, they may be useful alternative biocontrol agents. Takahashi *et al.* (2008) reported the biological characteristics of NPVs infectious to *A. honmai* larvae [12]. The killing speeds of AdhoNPV (Japanese isolate) and AdorNPV (English isolate) against neonate larvae were 19 and 6 days. On the other hand, the yield of AdhoNPV OBs was significantly higher than that of AdorNPV OBs. However, field studies of these isolates have not yet been conducted.

In this study, we examined two important parameters of AdhoNPV and AdorNPV as biocontrol agents: field control efficacy and viral transmission. The potential of these two slow- and fast-killing NPVs as biocontrol agents against *A. honmai* is discussed.

## 2. Materials and Methods

### 2.1. Insects

*A. honmai* used in experiments for speed of kill and cadaver location was obtained from the Agro-Kanesho Co., Ltd. (Tokorozawa, Saitama, Japan). To avoid genetic pollution of local *A. honmai* population, *A. honmai* released for the field trial in Shizuoka, were originated from Shimada, Shizuoka Prefecture, Japan. Both insect cultures were maintained in the laboratory at 25 °C, and with a 16-h light/8-h dark (16L: 8D) photoperiod. Larvae were reared on an artificial diet (Insecta LFS; Nosan Corporation, Yokohama, Kanagawa, Japan).

### 2.2. Viruses

AdhoNPV was originally isolated from diseased larvae collected in a tea field in Tsukuba, Ibaraki, Japan [8,13]. AdorNPV was originally isolated from diseased larvae collected in Kent, England [11]. Both viruses were propagated in *A. honmai* larvae and the OBs were purified as described elsewhere [9,10]. For the field trial, viral OBs were semi-purified as follows. Infected *A. honmai* cadavers were homogenized with distilled water. This suspension was filtered through two layers of gauze and centrifuged at 100× *g* for 5 min. The supernatant was centrifuged for 20 min at 3500× *g*, and the pellet was suspended in distilled water. The concentration of OBs in this stock suspension was determined by phase-contrast microscopy at 600× using a bacterial counter (Helber bacteria counting chamber, Hawksley, Sussex, UK), and the OBs were stored at −18 °C until used.

### 2.3. Killing Speed of AdhoNPV and AdorNPV

Larval *A. honmai* were inoculated with AdhoNPV or AdorNPV using a modified droplet feeding method [9]. Neonates and newly molted larvae were allowed to feed on a liquid inoculum containing a dose of >LC_95_ of AdhoNPV or AdorNPV OBs, 10% (*w*/*v*) sucrose, and 5% (*w*/*v*) red food coloring. The concentrations of inoculum were 3.0 × 10^8^ OBs/mL for neonate, 2^nd^ and 3^rd^ instar, 1.0 × 10^8^ OBs/mL for 4^th^ instar and 2.0 × 10^9^ OBs/mL for 5^th^ instar. The same solution without virus was used as a mock inoculum in control experiments. Larvae were reared until they died or pupated. The intervals at mortality assessments were made every 24 h.

### 2.4. Field Cage Trials

Field trials were conducted in three insect-proof net cages (4.5 × 4.5 × 2 m height) that were placed over two rows (1.5 × 3.7 × 0.7 m height) of tea plants (cv, “Yabukita”), in NARO Institute of Vegetable and Tea Science at Shimada, Shizuoka, Japan (34°48′ N, 138°08′ E). These three cages were used for two viral treatments (AdhoNPV and AdorNPV) and a virus-free control treatment.

Thirty egg masses, which had developed to the pharate larval stage (denoted by a change in the surface color to black), were transferred to each cage and attached to young leaves on 22 August 2008. Four days later, 0.2 L/m^2^ of OB suspension with 0.01% spreading agent “Gramin S” (Sankyo Agro, Tokyo, Japan) was applied to each cage using an electric sprayer (BH-593, Matsushita, Tokyo, Japan) with a single nozzle. The OB concentrations of AdhoNPV and AdorNPV were 4.4 × 10^9^ and 7.4 × 10^8^ OBs/m^2^, which yielded 90% mortality in a preliminary leaf dipping test. The control treatment cage was sprayed with water containing 0.01% Gramin S. The experiment was conducted only once.

**Figure 1:**
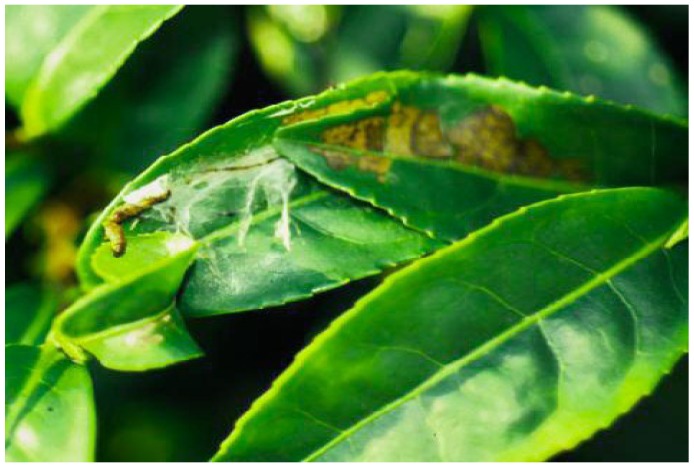
Rolled leaves, referred to as nests of *A. honmai* larvae. In the nest the larva damages the leaf. In this picture a cadaver is located outside its nest.

*A. honmai* larvae roll tea leaves and damage them in the rolled leaves, which are referred to as “nests” (Figure 1). Usually, a nest is produced by a single larva, and the number of nests is, thus, equivalent to the number of larvae. The number of nests and the size of the damage area on the surface of the leaves were investigated weekly by a quadrat method, at 1–3 weeks after spraying. This method consisted of randomly placing ten 0.5 × 0.5 m quadrats on the upper surface of tea plants in each cage on each assessment day, and recording the number of nests and the damage area of leaves in each square. The damage areas were categorized as “Small (<25 mm^2^)”, “Medium (25–100 mm^2^)”, “Large (100–400 mm^2^)”, or “Extra-large (>400 mm^2^)”.

At days after treatment, *A. honmai* 2^nd^ instar larvae were released into each cage to examine viral transmission to the next generation. The release timing of second generation was supposed as the timing of next generation of healthy *A. honmai* survived from viral application and/or migrated from other tea fields. Second release was done once and the timing (29 September 2008) was selected on the basis of data for developmental zero and thermal constants of healthy *A. honmai* [14]. Three hundred eighty larvae were released on four spots in each cage (1520 larvae per cage). Four days later, the released larvae were collected from each cage; these larvae were 2^nd^ or 3^rd^ instars. Larvae were transferred individually into 20-mL cups containing fresh artificial diet (Insecta LFS) and kept at 25 °C and a 16 L: 8D photoperiod in an incubator. Larvae were reared until they died or pupated. Dead insects were diagnosed as virus-infected if OBs were visible in tissue smears under a phase-contrast microscope. 

### 2.5. Position of Infected Insect Cadavers

Virus suspensions were prepared with OBs of AdhoNPV (2.2 × 10^7^ OBs/mL) and AdorNPV (3.7 × 10^6^ OBs/mL), equivalent to LC_90_ of neonate larvae in the droplet feeding method [12], and a final concentration of 0.02% of the spreading agent. Twenty milliliters of virus suspension was sprayed onto a branch of a tea plant with 30–40 leaves in Tokyo University of Agriculture and Technology in Fuchu, Tokyo, Japan (35°41′ N, 139°29′ E), which was then completely covered with a polyester net (22 cm diameter, 33 cm length, 1 mm mesh). A control branch was sprayed with 0.02% spreading agent. Spraying was done in the evening to avoid inactivation by sunlight, and each treatment was repeated six times. Egg masses of *A. honmai* were stapled to the underside of leaves, after the leaves were air-dried. Infected cadavers were collected 10 and 31 days after virus application for AdorNPV and AdhoNPV, respectively, and the number of insects that died at each of three positions (inside or outside rolled leaves, or having fallen off) was counted. NPV infection was confirmed by light microscopy.

### 2.6. Statistical Analysis

The time taken by viruses to kill the larvae was analyzed by nonparametric comparisons for two pairs using the Wilcoxon Test. The number of nests was log(x + 1) transformed and analyzed using analysis of variance (ANOVA). When there was a significant difference among treatments, Tukey-Kramer multiple comparison tests were performed (α = 0.05). Transmission rate and cadaver location data were arcsin square-root transformed and analyzed by *t*-test.

## 3. Results and Discussion

### 3.1. Killing Speed

**Figure 2:**
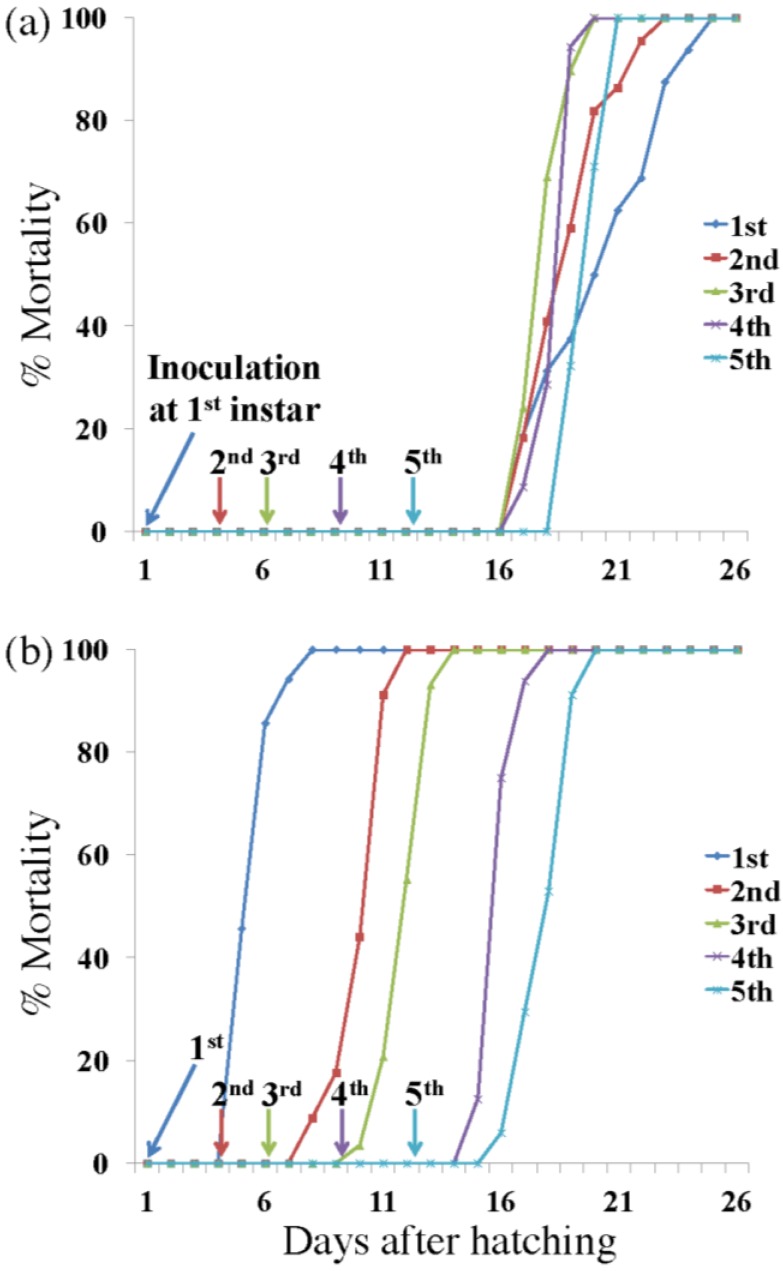
(**a**) Percentage mortality of *A. honmai* larvae inoculated with AdhoNPV at 1^st^, 2^nd^, 3^rd^, 4^th^ and 5^th^ instar; (**b**) Percentage mortality of *A. honmai* larvae inoculated with AdorNPV at 1^st^, 2^nd^, 3^rd^, 4^th^ and 5^th^ instar.

Figure 2 shows mortality and developmental time of AdhoNPV- and AdorNPV-infected *A. honmai*. AdhoNPV-infected larvae died only at the final instar regardless at which instar they were inoculated, and the mean survival time ± standard error (SE) was 18.9 ± 0.6, 15.2 ± 0.4, 12.2 ± 0.2, 10.1 ± 0.2, and 8.0 ± 0.1 days for neonate, 2^nd^, 3^rd^, 4^th^ and 5^th^ instar inoculation, respectively. On the other hand, the mean survival time ± SE of AdorNPV-infected larvae was 5.8 ± 0.1, 7.0 ± 0.1, 7.0 ±0.1, 8.0 ± 0.1, and 6.9 ± 0.1 days for neonate, 2^nd^, 3^rd^, 4^th^ and 5^th^ instar inoculation, respectively. Death caused by AdhoNPV infection was significantly slower than that by AdorNPV for any time of inoculation (1^st^ inoculation: *H* = 39.17, *p* < 0.01; 2^nd^ inoculation: *H* = 55.40, *p* < 0.01; 3^rd^ inoculation: *H* = 65.72, *p* < 0.01; 4^th^ inoculation: *H* = 55.03, *p* < 0.01; and 5^th^ inoculation: *H* = 21.02, *p* < 0.01).

The genomes of AdhoNPV and AdorNPV have been fully sequenced and compared with each other [11,13]. Among 121 putative ORFs, 118 are homologous to ORFs in AdhoNPV genome with 83%–100% amino acid identity. The main difference between them is partial deletion of *ecdysteroid UDP-glucosyltransferase* (*egt*) for AdorNPV compared to full possession of *egt* for AdhoNPV; however, genes related to killing speed of these viruses have not yet been determined. Differences in killing speed among closely related baculovirus genotypes have also been addressed in other baculovirus species. For instance, field isolates of *Agrotis ipsilon* NPV consist of isolates with and without an *egt* gene, and *egt*-deleted isolates showed decreased pathogenicity and increased killing speed [15]. *Spodoptera frugiperda* multiple NPV field isolates also include a fast-killing genotype that displays a deletion of *ORF sf27* and *egt*. Further study is needed to confirm *egt* or the other gene(s) involved in different killing speeds for AdhoNPV and AdorNPV. The two isolates were used to examine further whether killing speed of field isolates affects efficacy and viral transmission rate.

### 3.2. Field Efficacy within Sprayed Populations

**Figure 3:**
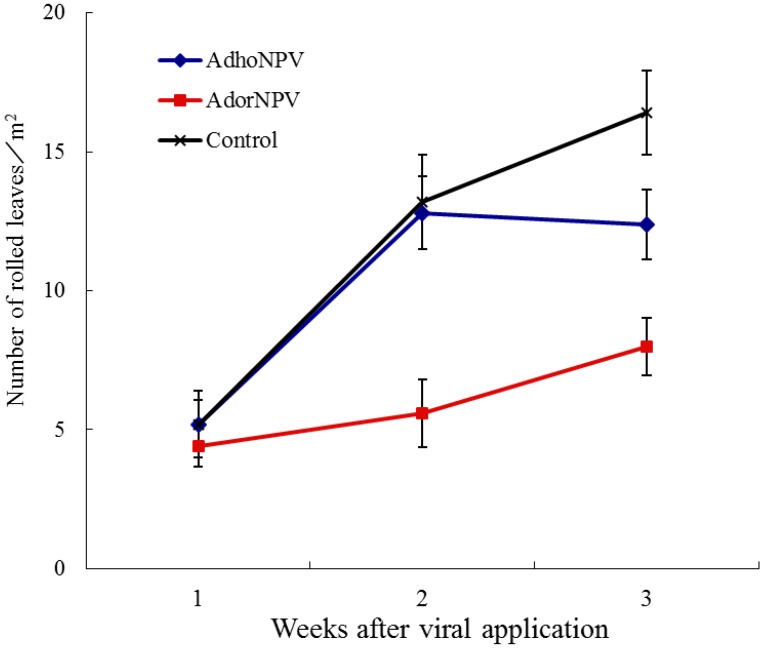
Number of rolled tea leaves produced by *A. honmai* larvae on tea plants in cages sprayed with AdhoNPV, AdorNPV, or virus-free control. Bars indicate standard errors.

AdorNPV showed greater potential than AdhoNPV for faster suppression of an *A. honmai* population. The mean numbers of *A. honmai* nests per quadrat for AdorNPV treatment were significantly lower than those for other treatments at two weeks (*F* = 8.47; *df* = 2, 27; *p* < 0.01) and three weeks after virus application (*F* = 10.79; *df* = 2, 27; *p* < 0.01), whereas there were no significant differences at one week (*F* = 0.09; *df* = 2, 27; *p* = 0.91) (Figure 3).

**Figure 4:**
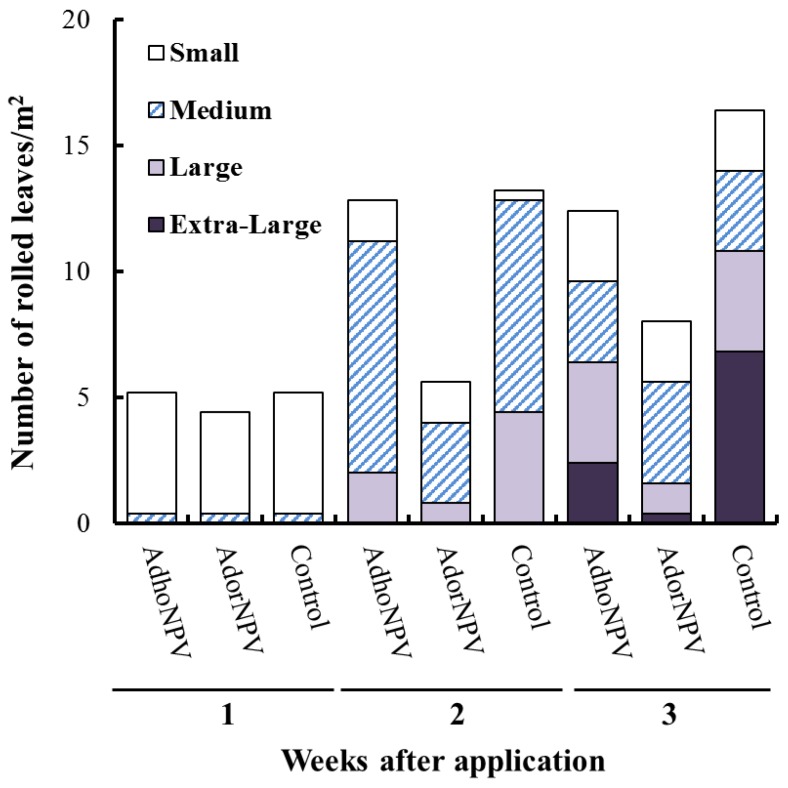
Number of rolled leaves produced by *A. honmai* larvae with damaged area. Damaged areas on the leaves were categorized as “Small (<25 mm^2^)”, “Medium (25–100 mm^2^)”, “Large (100–400 mm^2^)”, or “Extra-large (>400 mm^2^)”.

Damage areas in the treated plots were categorized to “Small”, “Medium”, “Large” and “Extra-Large” and the number of nests with damaged area were shown in Figure 4. The number of nests was less at 1 week because only visible nests were counted, and no significant difference was observed for “Small” and “Medium” damages among the treatment (Small, *F* = 0.0018, *df* = 2, 27, *p* = 0.998; Medium, *F* = 0.00, *df* = 2, 27, *p* = 1). At two weeks, more nests with “Large” damage were observed in the control and AdhoNPV-treatment than in AdorNPV, which was significantly different, as well as the number of “Medium” damages (Medium, *F* = 4.22, *df* = 2, 27, *p* = 0.0025; Large, *F* = 3.48, *df* = 2, 27, *p* = 0.045). At three weeks, damage areas in the control and AdhoNPV-treated cages were more than those in AdorNPV-treated cage. The number of nests in the “Large” and “Extra-large” categories in the control and AdhoNPV treatments were more than 50% of the total. In contrast, most of the nests in the AdorNPV treatment were “Small” (30%) and “Medium” (50%) (Figure 4). Number of leaves with “Small”, “Medium”, or “Large” damage were not significantly different among the three treatment (“Small”, *F* = 0.012, *df* = 2, 27, *p* = 0.98; “Medium”, *F* = 0.456, *df* = 2, 27, *p* = 0.64; “Large”, *F* = 2.25, *df* = 2, 27, *p* = 0.13), but number of leaves with “Extra-large” damage was significantly different (*F* = 11.80, *df* = 2, 27, *p* = 0.0002). Tukey-Kramer multiple comparison among the three treatments revealed that number of leaves with “Extra-Large” damage in control was significantly more than that in either AdhoNPV or AdorNPV (*p* < 0.05). Even though no significant difference was detected, number of leaves with “Extra-Large” damage was six-fold less in AdorNPV than in AdhoNPV (*p* > 0.05). These results were in agreement with previous laboratory bioassays [12].

The difference in efficacy between AdorNPV and AdhoNPV was probably due to the major difference in killing speed: the mean survival time of AdorNPV-infected larvae was six days, 13 days shorter than AdhoNPV-infected larvae (mean survival time: 19 days) when *A. honmai* larvae were inoculated at first instar (Figure 2). Speed of kill is the most important characteristic for insecticidal efficacy in the context of preventing crop damage.

Several baculoviruses have already been genetically engineered to improve their killing speed, a notable example being the insertion of an insect-specific toxin gene (AaIT) derived from the scorpion *Androctonus australis* (Scorpiones: Buthidae) into the baculovirus genome [2,16,17], and their superior efficacy has also been demonstrated in field trials [18,19,20]. Our results indicated that the fast-killing AdorNPV had superior potential to prevent damage to tea leaves, in comparison to the slow-killing AdhoNPV, against an *A. honmai* population during the generation in which the virus is applied.

### 3.3. Viral Transmission between Generations

**Figure 5:**
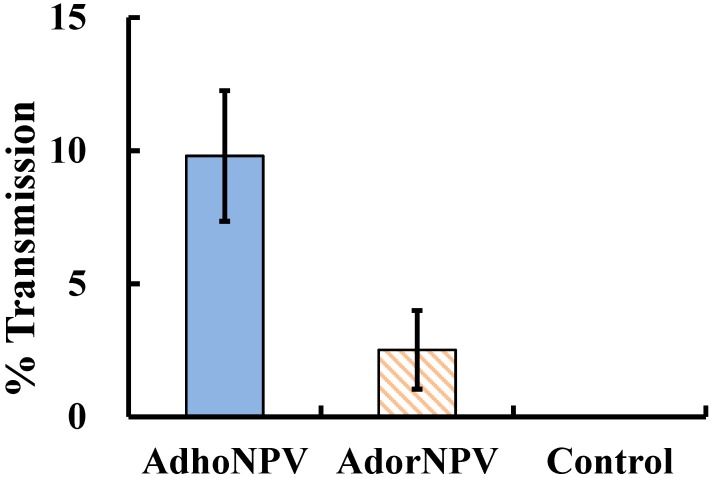
Transmission rate of AdhoNPV and AdorNPV to *A. honmai* larvae in the next generation. Percentage mortality of *A. honmai* larvae released at 34 days after spraying AdhoNPV and AdorNPV on tea leaves. A solution of spreading agent without virus was sprayed as the control. Bars indicate standard errors.

The transmission rate of AdhoNPV to *A. honmai* larvae was calculated as the percentage infection of larvae released 34 days after viral application, a time which is equivalent to the emergence of the next generation, and was approximately four-fold higher than that of AdorNPV (mean mortality of larvae recovered from AdhoNPV-treated cage = 9.80 ± 2.46; of those from AdorNPV-treated cage = 2.52 ± 1.48; *t* = 2.601; *df* = 6, *p* = 0.0406) (Figure 5). No viral transmission occurred in the control cage. No replication for viral application was done for this experiment. The difference between AdhoNPV and AdorNPV transmission may arise from variation in OB production for the two viruses: the yield of AdhoNPV OBs per larva (7.0 × 10^9^) was 1,000- to 10,000-fold higher than that of AdorNPV OBs (5.6 × 10^5^–8.7 × 10^6^; depending on instar at death) when neonate larvae were inoculated [12]. The amount of OB in a host habitat plays an important role in transmission among hosts [21,22]. 

**Figure 6:**
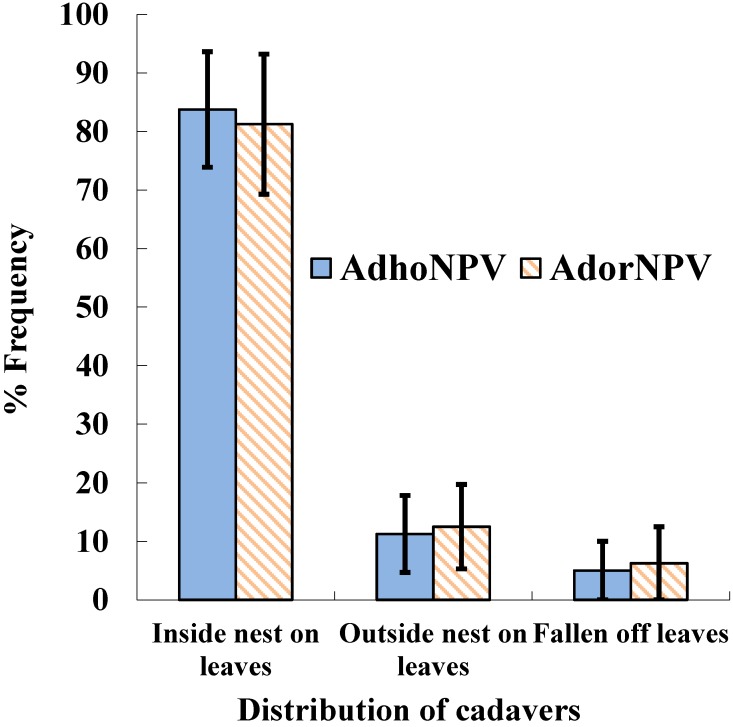
Location of *A. honmai* cadavers killed by AdhoNPV and AdorNPV on tea leaves. Bars indicate standard errors

Baculovirus infection is known to alter insect behavior, and several viral genes are implicated in “tree top disease” or “Wipfelkrankheit”, in which infected insects climb up the tree canopy and die near the top [23]. This phenotype is adapted for baculoviruses to increase their rate of transmission to other individual hosts. One of the genes involved in this behavior alteration caused by baculovirus infection is *egt*, because *Lymantria dispar* (Lepidoptera: Erebidae) larvae infected with an *egt* deletion mutant of *L. dispar* NPV climb less high than those infected with wild type virus [24]. Other genes and mechanisms also appear to be involved in this phenotype [25,26,27,28]. AdorNPV lacks functional domains in *egt*, whereas AdhoNPV possesses intact *egt*. However, both AdhoNPV- and AdorNPV-infected larvae died in their nest (Figure 6): 83.8% and 81.3% of cadavers killed by AdhoNPV and AdorNPV, respectively, were located inside nests on the leaves. There was no significant difference, for any position of infected insect cadavers, between AdhoNPV and AdorNPV (*p* > 0.05). Thus, the difference in transmission rate in our results was not attributable to the locations of infected cadavers, and the difference in OB production is likely to be a factor that drives the difference in transmission rates between the two isolates.

Hochberg (1989) categorized the location of virus-infected cadavers as either a “protected stage” or a “transmissible stage” [22]. According to a study of AaIT expressed by recombinant *Autographa californica* multiple NPV (AcMNPV), the transmission rate of the fast-killing recombinant virus is lower than that of the wild type AcMNPV, because the yield of recombinant virus was lower than that of wild type and because recombinant-infected larvae fell onto the soil before death [18,29]. Liquefaction of infected cadavers is known to enhance horizontal transmission of NPVs and the *v-cath* and *chi* genes, encoding *cathepsin* and *chitinase*, respectively, are involved in this process [30,31,32]. However, the AdhoNPV and AdorNPV genomes do not possess a *chi* gene, and infected cadavers do not liquefy, in contrast to other NPVs including AcMNPV [11,13]. Thus, progeny OBs from both AdhoNPV- and AdorNPV-infected larvae are in the “protected stage” until the cadavers are degraded by desiccation and spread to the tea leaf surface. Meanwhile, the rolled-leaf nests produced by *A. honmai* larvae may function as a reservoir of OBs, protecting the OBs from direct UV exposure until neonates (the most susceptible stage) of the subsequent generation arrive and are efficiently infected with virus. As mentioned previously, *A. honmai* have four to five discrete generations per year in Japan, and a slow-killing phenotype, such as AdhoNPV, may be more successfully transmitted to the next generation than a fast-killing phenotype. Other viruses that are infectious for *A. honmai* including AdorGV and AHEV also have a slow-killing phenotype, and the slow-killing trait in these pathogens may, therefore, be convergently adapted to the leaf roller system.

## 4. Conclusions

The results of this study revealed that the fast-killing isolate AdorNPV led to less damage to tea leaves than the slow-killing isolate AdhoNPV, since the slow-killing isolate showed a higher transmission rate than the fast-killing isolate; this is because the slow-killing isolate produced more progeny OBs than the fast-killing isolate. There are four main approaches used in microbial control of insects: the short-term microbial insecticide approach known as “inundative release”, the recycling microbial insecticide approach of “inoculative release”, the introduction-establishment approach, and environmental manipulation [33]. Takahashi *et al.* (2008) proposed that AdorNPV is appropriate for inundative release, while AdhoNPV is appropriate for inoculative release [12]. This study has demonstrated that the fast-killing AdorNPV controls pests more efficiently in the short term than the slow-killing AdhoNPV because of its higher field efficacy against pests within the generation exposed to virus. On the other hand, the slow-killing AdhoNPV controls pests more effectively than fast killing AdorNPV in the long term because of its higher transmission efficiency. AdhoNPV may have an advantage in field efficacy against the sprayed population compared to the commercial agent Hamaki-Tenteki, which includes AdorGV, because the killing speed of AdhoNPV is faster than that of AdorGV. Further field experiments on a larger scale are necessary to evaluate the long-term control efficiency of AdhoNPV as a candidate alternative control agent to AdorGV.
